# Parathyroid Carcinoma: From Molecular Pathogenesis and Diagnostic Biomarkers to Targeted Therapeutics

**DOI:** 10.3390/ijms27104549

**Published:** 2026-05-19

**Authors:** Chunlong Wang, Xiaoqing Wu, Yuqin Liu

**Affiliations:** 1 Cell Resource Center, Department of Pathology, Institute of Basic Medical Sciences, School of Basic Medicine, Chinese Academy of Medical Sciences (CAMS), Peking Union Medical College (PUMC), Beijing 100005, China; 15949860779@163.com; 2 Geriatric Department, Peking University People’s Hospital, Beijing 100044, China; wuxq0611@163.com

**Keywords:** parathyroid carcinoma, CDC73, parafibromin, molecular pathology, epigenetic reprogramming, targeted therapeutics, biomarker

## Abstract

Parathyroid carcinoma (PC) is a rare endocrine malignancy characterized by aggressive clinical behavior driven primarily by parathyroid hormone (PTH) overproduction. Standard morphological assessments frequently struggle to definitively distinguish true carcinomas from atypical benign lesions, presenting significant diagnostic challenges and a risk of overdiagnosis. Recent advances emphasize the genetic and epigenetic foundations of PC tumor biology. A central oncogenic mechanism involves the CDC73 gene, where the biallelic inactivation of CDC73 and the gain of function of mutant parafibromin—which destabilizes p53 mRNA—facilitate apoptosis evasion. Additionally, alterations in parallel pathways, such as the PI3K/AKT/mTOR cascade, and epigenetic dysregulation further contribute to disease progression. To address morphological limitations, contemporary diagnostic approaches increasingly utilize adjunctive multimarker immunohistochemical panels (including parafibromin, Ki-67, and Galectin-3) and explore emerging non-coding RNA liquid biopsy signatures. Finally, this review discusses the development of preclinical models and the application of genotype-guided targeted therapies, aiming to improve the clinical management and precision medicine strategies for PC.

## 1. Introduction

Parathyroid carcinoma (PC) is a rare and devastating endocrine malignancy, accounting for approximately 1% of primary hyperparathyroidism cases worldwide [1]. Its highly aggressive clinical profile is primarily driven by profound parathyroid hormone (PTH) overproduction, rather than just the tumor burden [2]. This hypersecretion induces intractable hypercalcemia and severe multi-organ toxicity [3]. Furthermore, PC frequently presents with atypical clinical manifestations that obscure the initial diagnosis. For example, ectopic PTH secretion from nonparathyroid tissues and mediastinal tumors creates formidable surgical and localization challenges [4,5]. These atypical presentations provide critical insights into cancer endocrinology [6]. Although PC typically progresses slowly, clinical evidence from large population-based cohorts highlights its life-threatening potential. According to large-scale analyses, such as the SEER database cohort, the 5-year and 10-year overall survival (OS) rates for PC range from 82 to 85% and 49 to 77%, respectively [7]. Notably, the prognosis significantly worsens for patients presenting with distant metastasis, whose 5-year survival rate drastically drops to less than 50% [2,7].

Diagnosing PC remains a profound dilemma in endocrine surgical pathology. Some investigators debate whether insidious presentations mask the true incidence of parathyroid malignancies, suggesting potential underdiagnosis [8]. Conversely, strict warnings emphasize the critical need to avoid overdiagnosing PC. Pathologists must note that mechanical artifacts, prior biopsies, or iatrogenic fibrosis can deceptively simulate true capsular or vascular invasion [9]. Because standard morphological assessments often fail to resolve borderline cases, contemporary diagnostic algorithms increasingly rely on vital adjuncts [10]. Specifically, comprehensive molecular imaging [11,12] and strategic immunohistochemistry [13] are now indispensable for distinguishing true carcinomas from atypical benign lesions.

Given these morphological limitations, the field is reevaluating how parathyroid malignancies are classified and managed [14]. Consequently, parathyroid oncology is shifting from standard clinicopathological evaluations toward detailed genetic and epigenetic analyses of tumor biology. These insights highlight the central oncogenic roles of the biallelic inactivation of CDC73 [15,16] and targeted promoter hypermethylation [17,18]. Furthermore, single-cell transcriptomic profiling has unmasked intratumoral heterogeneity, revealing complex interactions between malignant endocrine cells and an immunosuppressive microenvironment [19]. Together, these discoveries radically reshape our biological understanding of parathyroid tumorigenesis [20].

Building upon this foundation, this review aims to bridge the translational gap in parathyroid oncology [21]. By synthesizing recent advances in clinical management [7], we systematically navigate the genomic, epigenetic, and molecular pathology landscapes of PC. Ultimately, by mapping these molecular vulnerabilities, we explore the transition toward robust preclinical models and genotype-guided targeted therapies, establishing a clear roadmap for precision medicine in PC.

## 2. Molecular Pathogenesis of PC

**Figure 1:**
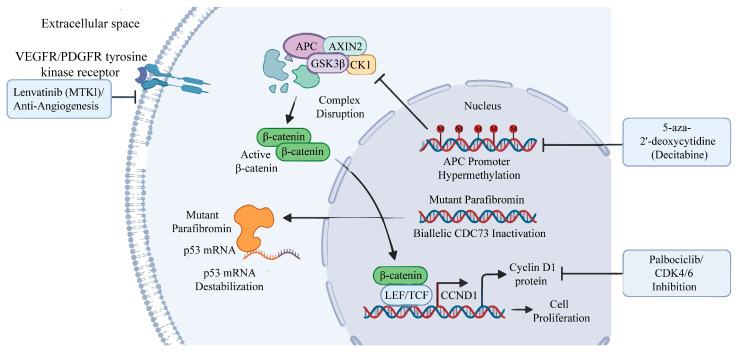
**Comprehensive molecular pathogenesis and genotype-guided targeted therapeutics in parathyroid carcinoma (PC).** The schematic illustrates the complex interplay of epigenetic silencing, genetic mutations, and aberrant signaling pathways, alongside their corresponding precision medicine interventions. (Nucleus and Cytoplasm) Within the nucleus, aberrant promoter hypermethylation silences the tumor suppressor gene APC. This epigenetic event leads to the physical disassembly of the cytoplasmic β-catenin destruction complex (composed of APC, AXIN2, GSK3β, and CK1). Consequently, non-phosphorylated, active β-catenin accumulates and translocates into the nucleus, where it docks with LEF/TCF transcription factors to drive the robust transcription of oncogenic targets, including CCND1, ultimately promoting cell proliferation. Concurrently, the biallelic inactivation of CDC73 generates mutant parafibromin, which translocates to the cytoplasm and exerts a toxic gain of function by actively binding to and destabilizing p53 mRNA, effectively facilitating apoptosis evasion. (Targeted Therapeutics) The diagram highlights three critical genotype-guided pharmacological interventions: (1) Decitabine (5-aza-2′-deoxycytidine) acts to reverse APC promoter methylation, offering a mechanistic strategy to reconstitute the destruction complex. (2) Palbociclib provides targeted CDK4/6 inhibition against the overexpressed Cyclin D1 protein, arresting the cell cycle. (3) At the cell membrane, Lenvatinib, a multitarget tyrosine kinase inhibitor (MTKI), blocks VEGFR/PDGFR receptors to strongly suppress tumor angiogenesis. (Abbreviations: PC, parathyroid carcinoma; APC, adenomatous polyposis coli; GSK3β, glycogen synthase kinase 3 beta; CK1, casein kinase 1; LEF/TCF, lymphoid enhancer-binding factor/T-cell factor; CCND1, cyclin D1; MTKI, multitarget tyrosine kinase inhibitor).

The clinical aggressiveness of PC is rooted in a complex interplay of genomic instability and epigenetic dysregulation. The central molecular landscape, integrating the biallelic inactivation of tumor suppressors, aberrant activation of signaling cascades, and potential therapeutic nodes, is systematically illustrated in Figure 1.

### 2.1. Genomic Alterations of the CDC73 Axis

The CDC73 gene (formerly HRPT2), located on chromosome 1q31.2, is the predominant tumor suppressor gene implicated in the pathogenesis of PC. Germline heterozygous loss-of-function mutations in CDC73 are the genetic foundation of Hyperparathyroidism–Jaw Tumor (HPT-JT) syndrome, occurring in 50% to 75% of these pedigrees, and are also found in approximately 8% of families with familial isolated primary hyperparathyroidism (FIHP) [22]. In the non-syndromic (sporadic) setting, the CDC73 mutational burden remains the primary oncogenic driver. Occult CDC73 germline mutations are identified in 20% to 40% of patients presenting with apparently sporadic PC, frequently unmasking previously unrecognized HPT-JT syndrome [23].

Furthermore, somatic CDC73 mutations are reported to occur in roughly 70% (ranging from 40% to 100% depending on the series) of sporadic PC cases, but they are exceedingly rare in benign parathyroid adenomas [24,25]. Tumorigenesis classically adheres to Knudson’s “two-hit” hypothesis, requiring biallelic gene inactivation for malignant transformation. Demonstrating this mechanism, Cardoso et al. [24] documented that somatic loss of heterozygosity (LOH) involving the 1q31.2 CDC73 locus occurs in 50% to 55% of sporadic PC cases. The specific mutational genotype profoundly dictates the clinical risk; an extensive analysis of 419 individuals conducted by Li et al. [26] revealed that carriers of “high-impact” CDC73 germline variants—specifically gross deletions, frameshift indels, splice-site disruptions, and stop-gain mutations—face a greater than 10-fold higher risk of developing PC compared to individuals harboring low-impact missense variants.

### 2.2. Parafibromin, the PAF1 Complex, and Transcriptional Control

The CDC73 gene encodes parafibromin, an evolutionarily conserved 531-amino acid protein normally localized to the cell nucleus. At the molecular level, parafibromin functions as an essential scaffold component of the human Polymerase-Associated Factor 1 (PAF1) complex [22]. By directly associating with RNA polymerase II, the PAF1 complex orchestrates critical nuclear events, including regulation of gene transcription, elongation of gene transcripts, and mRNA 3′-end processing.

Beyond transcript elongation, parafibromin and menin exert profound epigenetic control over chromatin architecture by interacting directly with the histone methyltransferase SUV39H1 [24,27]. Through this interaction, parafibromin functions as a potent transcription repressor by inducing the methylation of histone H3 at lysine 9 (H3K9me). In addition to its epigenetic functions, wild-type parafibromin acts as a critical negative regulator of the canonical Wnt/β-catenin signaling cascade. As documented by Cardoso et al. [24] and Juhlin et al. [28], wild-type parafibromin physically associates with β-catenin, heavily influencing and stringently repressing the transcription of specific downstream pro-oncogenic targets, most notably the c-Myc proto-oncogene and the CCND1 gene. Consequently, following the biallelic inactivation of the CDC73 gene, this suppression is catastrophically lifted, leading to aberrant gene expression and cellular proliferation.

### 2.3. The Toxic Gain of Function: Mutant Parafibromin and Apoptosis Evasion

While parathyroid carcinogenesis is heavily dependent on the biallelic inactivation of CDC73, specific CDC73 mutations actively precipitate malignancy through toxic gain-of-function mechanisms that cripple cellular apoptotic responses. Aligning directly with the oncogenic mechanisms illustrated in Figure 1, recent analyses [29] have elucidated this mechanism by demonstrating that the mutant form of parafibromin actively binds to and destabilizes p53 mRNA.

Under normal physiological conditions, p53 acts as the master regulator of apoptosis in response to cellular stress and DNA damage. By aberrantly binding to and destroying p53 transcripts, the mutant form of parafibromin essentially neutralizes p53-mediated apoptosis. The evasion of this critical apoptotic checkpoint confers a profound survival advantage to CDC73-mutated parathyroid cells, directly fueling continuous malignant expansion and preserving heavily mutated, genomically unstable cellular clones.

### 2.4. CCND1 Amplification and Cell Cycle Dysregulation

The CCND1 gene (also known as PRAD1), located on chromosome 11q13.3, acts as a highly potent oncogene in parathyroid carcinogenesis. It encodes Cyclin D1, an indispensable positive regulator of the cell cycle. Cyclin D1 overexpression is a major molecular feature of PC, detected in 65% to 90% of malignant parathyroid tumors [24,30]. Once overexpressed, Cyclin D1 forms an active complex with cyclin-dependent kinase 4 (CDK4), which hyper-phosphorylates the retinoblastoma 1 (RB1) tumor suppressor protein. This neutralizes RB1’s ability to inhibit the G1-to-S phase transition, leading to uncontrolled cellular proliferation.

The intense overexpression of CCND1 in PC is driven by a convergence of genomic amplification and Wnt pathway hyperactivation. Genomic studies reveal that a two- to three-copy number gain of the CCND1 gene occurs in approximately 70% of PC cases [31], directly correlating with elevated CCND1 mRNA and protein levels. Synergistically, as illustrated in the mechanism in Figure 1, CCND1 transcription is robustly driven by Wnt signaling and pathological β-catenin nuclear translocation. This β-catenin accumulation is severely exacerbated by upstream epigenetic anomalies. Hypermethylation of the APC promoter 1A abolishes the expression of the APC protein in roughly 75% of PCs [32]. Because APC normally functions in the destruction complex to degrade β-catenin, its epigenetic silencing results in massive cytoplasmic accumulation and subsequent nuclear translocation of active, non-phosphorylated β-catenin. Furthermore, overexpression of the EZH2 histone methyltransferase epigenetically represses Wnt antagonists like AXIN2, compounding β-catenin accumulation [33]. Ultimately, this hyperactive Wnt/β-catenin cascade drives massive, unrestrained CCND1 transcription [34].

### 2.5. Emerging Mutational Drivers: FLCN and the PI3K/AKT/mTOR Cascade

Comprehensive genomic profiling has expanded the molecular landscape of PC to include other critical drivers, notably the FLCN gene and the PI3K/AKT/mTOR signaling cascade, which operate as parallel mutational pathways to the CDC73 axis. The FLCN (Folliculin) tumor suppressor gene, classically associated with Birt–Hogg–Dubé (BHD) syndrome, is increasingly implicated in parathyroid malignancy [35]. Recurrent somatic “hot spot” mutations (e.g., c.1285insC) cause frameshifts resulting in unstable, truncated proteins with lost tumor-suppressive capabilities [35,36]. Under physiological conditions, Folliculin interacts with FNIP1/FNIP2 to strictly regulate mTORC1 signaling; its inactivation triggers pathological mTORC1 activation [37].

Direct mutations within the PI3K/AKT/mTOR cascade further solidify its role as a master regulatory network driving PC. Somatic alterations constitutively activating this network are found in up to 20% of PC cases [16,38]. Targeted deep sequencing reveals actionable somatic variants in crucial pathway components in nearly 25% of samples [39], encompassing mutations in PIK3CA, PTEN, MTOR, TSC1, and TSC2 and novel truncating mutations in PIK3CB (e.g., p. Q75*) [40].

Clinically, activation of the PI3K/AKT/mTOR pathway dictates a highly aggressive disease phenotype, manifesting with substantially higher serum intact PTH levels and conferring significantly shorter disease-free and overall survival compared to patients lacking these aberrations [39]. Notably, these pathway alterations are often mutually exclusive with CDC73 mutations, presenting a distinct, highly aggressive molecular subset of CDC73-wild-type parathyroid carcinomas [41].

## 3. Epigenetic Reprogramming and the Tumor Immune Microenvironment

### 3.1. Methylation and Chromatin Modifiers

Epigenetic dysregulation, particularly aberrant DNA methylation, is now recognized as a fundamental mechanism driving parathyroid tumorigenesis and establishing malignant phenotypes. Comprehensive reviews by Silva-Figueroa and Perrier [42], as well as Miratashi Yazdi and Nazar [43], have emphasized that modifications in chromatin structure and global DNA methylation events are indispensable for initiating PCs. Supporting this, genome-wide methylome analyses by Starker et al. [44] demonstrated that PCs do not typically exhibit the massive global hypomethylation seen in other cancers; instead, they feature targeted, dense hypermethylation of specific CpG islands. This local hypermethylation effectively silences critical tumor suppressor genes and cell cycle regulators, including CDKN2A, CDKN2B, and WT1.

Crucially, the enzymatic regulation of DNA demethylation is also profoundly disrupted in PC. Investigations by Barazeghi et al. [45] revealed that the TET2 gene undergoes dense promoter hypermethylation in PCs, a mechanism that abolishes TET2 protein expression. Consequently, as synthesized in the surgical pathology overview by Juhlin and Erickson [28], alongside specific functional studies by Barazeghi et al. [45], PCs display a near-complete global depletion of the 5-hydroxymethylcytosine (5-hmC) epigenetic mark. This systemic failure of DNA demethylation appears to be an exclusive hallmark of malignant transformation in parathyroid tissues, offering a highly accurate marker for discriminating PCs from benign parathyroid adenomas (PAs).

### 3.2. Histone Modifications and WNT/β-Catenin Crosstalk

In PC, epigenetic alterations frequently converge to constitutively activate the WNT/β-catenin signaling axis. Pivotal in vitro studies by Svedlund et al. [32] demonstrated that the APC promoter 1A is heavily hypermethylated in PCs. This hypermethylation extinguishes the expression of the APC tumor suppressor, which physically disrupts the β-catenin destruction complex. As detailed by Westin [46], active, non-phosphorylated β-catenin subsequently accumulates in the cytoplasm and translocates to the nucleus, functioning as a central oncogenic “hub” in PC. This epigenetic bypass is critical, as large-scale genomic profiling by Pandya et al. [16] confirmed that direct somatic mutations in CTNNB1 are exceptionally rare in these tumors.

Histone modifiers further amplify this oncogenic cascade. Marini et al. [17] elucidated that EZH2 gene amplification (observed in approximately 60% of sporadic PCs) catalyzes repressive histone H3 lysine 27 trimethylation (H3K27me3). This specific chromatin modification effectively silences WNT antagonists such as AXIN2 and the HIC1 tumor suppressor, synergistically driving WNT transduction. Furthermore, the previously described loss of the parafibromin–SUV39H1 complex at the CCND1 promoter [47] directly eradicates H3K9 methylation defenses, ensuring uninhibited Cyclin D1 overexpression.

### 3.3. Dysregulation of the Non-Coding RNA Transcriptome

The parathyroid malignant landscape features extensive rewiring of the non-coding RNA transcriptome. Wielogórska et al. [48] noted that this process is generally characterized by a global downregulation of tumor-suppressive microRNAs (miRNAs) and the targeted upregulation of oncogenic transcripts. Initial profiling by Corbetta et al. [49] identified a distinct microRNA signature in CDC73-mutated PCs, marked by severe downregulation of miR-296 and miR-139, alongside overexpression of miR-222 and miR-503.

Expanding this transcriptomic map, Rahbari et al. [50] identified significant diagnostic downregulation of miR-126*, miR-26b, and miR-30b in PCs. Subsequently, Hu et al. [51] rigorously verified these candidate miRNA markers in an independent cohort, demonstrating that a combined diagnostic index of miR-139 and miR-30b achieves high clinical accuracy (AUC = 0.888) in separating PC from benign disease. Translating these findings into liquid biopsies, a pilot study by Krupinova et al. [52] demonstrated that circulating miR-342-3p levels are profoundly diminished in the serum of PC patients, offering a non-invasive avenue for preoperative risk stratification.

Beyond miRNAs, comprehensive microarray profiling by Zhang et al. [53] pinpointed the dramatic upregulation of the long non-coding RNA (lncRNA) PVT1 and the downregulation of GLIS2-AS1 in PC tissues. The diagnostic utility of lncRNAs is further bolstered by the findings of Morotti et al. [54], who established that circulating levels of the lncRNA BC200 are significantly elevated in PC patients, particularly those with metastatic disease, and drop precipitously following parathyroidectomy.

### 3.4. The Tumor Immune Microenvironment and Emerging Genetics

The integration of whole-exome sequencing (WES) has shifted the genetic paradigm of PC. Simonds [55] comprehensively reviewed the genetics of hyperparathyroidism, noting that WES of sporadic PCs uncovers immense genetic heterogeneity. Recent genomic studies investigating novel PRUNE2 germline mutations by Storvall et al. [56], alongside foundational WES data by Yu et al. [23], have expanded the mutational landscape to include defects in genes governing cell migration, as well as distinctive APOBEC-catalyzed DNA mutagenesis spectra. Furthermore, comprehensive multi-omic profiling has highlighted the constitutive activation of the PI3K/AKT/mTOR pathway—evidenced by mutations in PIK3CA and MTOR—as a hallmark of malignant severity and poorer survival in PC [39].

Despite this complex mutational load, the local tissue ecology of PC actively dictates disease progression through profound immunosuppression. Groundbreaking work by Silva-Figueroa et al. [57] classified PCs as predominantly “immune-ignorant” or “immune-tolerant,” defined by widespread negativity for PD-L1 and sparse lymphocyte infiltration. Quantifying this tumor immune microenvironment (TIME), Hu et al. [58] established that stromal immunocyte density in PC is directly correlated with disease relapse. Their cohort demonstrated that a heavy infiltration of CD163+ tumor-associated macrophages (M2 subtype), coupled with a severe depletion of direct cytotoxic CD8+ T cells, acts as an independent, highly significant prognostic indicator of postoperative PC recurrence. This immunosuppressive architecture underscores the urgent need to explore macrophage-depleting agents and targeted immunotherapies for refractory disease.

## 4. Molecular Pathology and Diagnostic Biomarkers

### 4.1. Morphological Challenges and the Risk of Overdiagnosis

The pathological diagnosis of PC remains heavily dependent on morphological evaluation, which serves as the undisputed gold standard [9]. The ultimate endpoint for any molecular marker is to guide the surgical pathologist in diagnostics and prognostication when morphology yields ambiguous results [28]. Many architectural features commonly associated with PC, such as dense fibrous bands and high mitotic activity, are not specific to malignancy and frequently overlap with those of atypical parathyroid tumors [28,59]. Thus, a definitive PC diagnosis strictly requires unequivocal evidence of invasive growth (such as angioinvasion, lymphatic invasion, perineural invasion, or direct invasion into adjacent structures) or documented metastasis [60,61].

Furthermore, there is a substantial risk of overdiagnosis resulting from histopathologic mimics; specifically, mechanical artifacts from prior fine-needle aspiration, ethanol injection, or parathyroid hormone (PTH) washout can induce severe fibrosis and pseudoinvasion [9]. Peliosis (extravasated erythrocytes lacking an endothelial lining) can also deceptively simulate true vascular invasion. Within this morphologically challenging landscape, immunohistochemistry serves as a vital adjunct [62]. Nevertheless, the isolated loss of parafibromin expression does not definitively establish malignancy, as parafibromin deficiency can also occur in morphologically benign adenomas (such as those associated with HPT-JT syndrome), making meticulous morphological correlation paramount [9,63].

### 4.2. The Cornerstone: Parafibromin and Cell Cycle Regulators

Translating the foundational CDC73 genomic alterations into clinical practice, the loss of its protein product [64] establishes parafibromin immunohistochemistry as a primary diagnostic surrogate marker [65]. However, its diagnostic performance as a standalone marker is imperfect. In a comprehensive meta-analysis of ten studies, Hu et al. [66] demonstrated that the pooled sensitivity of parafibromin loss for identifying PC was limited to 68% (95% CI: 49–82%), whereas its pooled specificity remained highly robust at 95% (95% CI: 85–98%). Therefore, retained parafibromin expression cannot reliably exclude malignancy [67]. Beyond diagnosis, the prognostic value of parafibromin is highly significant. In a retrospective cohort study of 107 parathyroid lesions, Hu et al. [62] established via Cox proportional hazards analysis that complete loss of parafibromin staining was an independent risk factor strongly associated with PC recurrence (hazard ratio: 3.26). Beyond their indispensable role in differential diagnosis, specific molecular biomarkers carry profound prognostic value in clinical settings. Clinical cohort studies and meta-analyses have consistently demonstrated that the global loss of parafibromin expression, along with the downregulation of CASR, acts as a strong negative determinant of prognosis [56,67]. These molecular signatures significantly correlate with shorter disease-free survival (DFS) and higher risk of postsurgical recurrence and mortality. To compensate for parafibromin’s limited sensitivity, the assessment of cell cycle regulators is routinely incorporated. Uljanovs et al. [65] highlighted that a high proliferative index, defined by Ki-67 expression exceeding 5%, is frequently upregulated in PC compared to benign parathyroid tissues. Furthermore, Erovic et al. [68] advocated for the evaluation of additional cell cycle inhibitors, demonstrating that the loss of retinoblastoma (RB) and p27 proteins can further aid in characterizing the malignant potential of challenging neoplasms.

### 4.3. Adjunctive Multimarker Panels

Because single biomarkers lack absolute diagnostic precision, contemporary endocrine pathology has shifted toward combinatorial multimarker panels. In an extensive evaluation of 227 parathyroid neoplasms, Kumari et al. demonstrated that the adjunctive markers Galectin-3 and PGP9.5 each yielded a low individual sensitivity of 45.4% for detecting PC but maintained high specificities of 90.2% and 85.0%, respectively. Furthermore, they noted that a combined profile featuring parafibromin loss alongside Galectin-3 and PGP9.5 overexpression achieved a 50% sensitivity and a highly reliable specificity of 97.9% [69]. To mathematically optimize these variables into a practical clinical tool, Silva-Figueroa et al. [70] evaluated a cohort of 63 patients and developed a novel diagnostic nomogram incorporating the continuous percentage expression of five markers: parafibromin, RB, PGP9.5, Ki-67, and Galectin-3. This combinatorial nomogram successfully discriminated PC from atypical and benign neoplasms, yielding a high area under the curve (AUC) of 0.849 (optimism-adjusted to 0.805). More recently, Hu et al. [62] expanded this multimarker strategy by examining 107 parathyroid lesions. They uncovered that membranous E-cadherin loss was present in 47.7% of PCs compared to only 18.6% of benign controls, while nuclear EZH2 positivity was observed in 31.8% of PCs but was entirely absent (0%) in the control cases. Ultimately, they validated a streamlined, three-marker logistic regression model incorporating parafibromin, Ki-67, and E-cadherin, which achieved a highly accurate diagnostic AUC of 0.843.

### 4.4. Emerging Epigenetic and Liquid Biopsy Signatures

The molecular landscape of PC is further characterized by profound epigenetic remodeling and the emergence of transcriptomic liquid biopsy signatures. Barazeghi et al. [71] investigated the epigenetic mark 5-hydroxymethylcytosine (5-hmC), a critical intermediate in active DNA demethylation [71]. In a strictly defined cohort consisting of 17 PCs and 43 benign adenomas, they observed a complete absence of 5-hmC immunostaining in all 17 PC cases, contrasted with positive retention in all 43 adenomas. While this specific limited cohort demonstrated a 100% sensitivity and 100% specificity for 5-hmC loss in distinguishing PC, these absolute metrics dictate that broad multi-center validation is required before universal clinical implementation can be recommended. In the rapidly evolving realm of liquid biopsies, Morotti et al. [54] identified the long non-coding RNA (lncRNA) BC200 as an overexpressed circulating oncogenic transcript in the serum of PC patients. Their study demonstrated that circulating BC200 levels can accurately discriminate PC from benign adenomas preoperatively and drop significantly following surgical resection, offering a novel, non-invasive biomarker for disease monitoring. Finally, in exceptionally rare and diagnostically challenging disease variants, such as non-functional parathyroid carcinoma, Jentus et al. noted that the absence of classic biochemical hyperparathyroidism markers necessitates an even heavier reliance on rigorous immunohistochemical and epigenetic profiling, utilizing these emerging signatures to definitively confirm the cellular origin and malignant nature of the silent cervical mass [72]. The key molecular and epigenetic biomarkers discussed above, along with their clinical utilities and levels of evidence, are systematically summarized in Table 1.

## 5. Overcoming the Translational Bottleneck: Preclinical Models, Targeted Therapeutics, and Future Directions

### 5.1. The Scarcity of Preclinical Models

The historical scarcity of stable in vitro and in vivo preclinical models remains a persistent bottleneck in understanding PC pathogenesis and accelerating therapeutic discovery [74]. Primary human parathyroid cells have a very short lifespan in the lab and quickly stop secreting PTH [7,75], making it difficult to test cancer-causing genes and targeted drugs. To address this, Gogusev et al. [74] successfully created a continuous human PC cell line. This immortalized cell platform is a major breakthrough, allowing for detailed molecular studies and high-throughput drug testing [19]. However, a critical limitation of current in vitro models is their inherent inability to accurately recapitulate the complex stromal and immune cell interactions of the native tumor microenvironment [76]. This crucial missing link frequently leads to the failure of preclinical drug efficacy in real-world human trials [7]. Overcoming this translational bottleneck requires the urgent establishment of immunocompetent in vivo models and advanced patient-derived organoids (PDOs) capable of faithfully recreating the 3D architecture and immune landscape of the original tumor to rigorously validate targeted therapies.

### 5.2. Multi-Omics Guided Vulnerabilities and Cell Cycle Inhibition

Next-generation sequencing has moved PC research from a focus on physical structure to the era of molecular oncology [77]. Genomic profiling has mapped the unique molecular signatures that separate PCs from benign tumors [78]. This has helped identify targetable cancer pathways. A universal feature of PC is the disruption of the cell cycle, often caused by CCND1 amplification and biallelic inactivation of CDC73 [79,80,81]. Therefore, targeting the Cyclin D1-CDK4/6-Rb pathway is a logical nonsurgical treatment. Testing this idea, preclinical models show that CDK4/6 inhibitors (e.g., palbociclib) strongly suppress tumor growth in PCs driven by CCND1 [81]. Future targeted therapies must rely on the specific molecular subtype of each patient [22]. Using tailored drugs for specific genetic defects, like CDC73 mutations, will solidify precision medicine for PC [22].

### 5.3. Tyrosine Kinase Inhibitors and Anti-Angiogenic Therapy

PC tumors usually have a dense blood supply [22,82]. This makes blocking new blood vessel growth an attractive treatment for unremovable or spreading disease. Recently, multitarget tyrosine kinase inhibitors (MTKIs) have shown great promise for palliative care [82,83]. For example, Ito et al. [82] successfully treated a metastatic PC patient with Lenvatinib. This MTKI strongly blocks blood vessel growth receptors like VEGFR and PDGFR. Molecular testing revealed that this patient’s tumor had a MEN1 mutation and high VEGFR levels [82]. As a result, Lenvatinib stabilized the metastases and controlled life-threatening high calcium levels. Supporting this approach, Teleanu et al. [77] used sequencing to find FGFR1 and RET overexpression in advanced PC. This further proved that Lenvatinib can stabilize the disease and restore biochemical balance. However, these targeted treatment responses are often temporary. Doctors must carefully manage the side effects of these drugs. To bridge the gap between preclinical models and patient care, emerging clinical evidence has highlighted the efficacy of targeted therapeutics and immunotherapies in advanced PC. Although large-scale Phase III clinical trials are inherently limited by the extreme rarity of this malignancy, multi-target tyrosine kinase inhibitors (MTKIs) such as Lenvatinib and Sorafenib have demonstrated clinical success in controlling refractory hypercalcemia and reducing tumor burden in metastatic patients [77]. Furthermore, precision medicine approaches using Temozolomide have achieved significant long-term clinical remission in advanced PC cases exhibiting high MGMT promoter methylation [84]. Most recently, clinical insights have also revealed the potential of immune checkpoint inhibitors, such as Pembrolizumab, in managing metastatic PC driven by microsatellite instability (MSI), shifting the therapeutic landscape towards personalized, real-world clinical interventions [20].

### 5.4. The Surgical Gold Standard and Future Perspectives

Despite promising drug advances, medical therapies for advanced PC are still mainly palliative [20,85]. Therefore, complete surgical removal at the time of diagnosis remains the only true cure [86]. The surgical gold standard requires an aggressive en bloc resection [87]. This means removing the tumor, the thyroid lobe on the same side, and any attached tissues to lower the high risk of recurrence.

Ultimately, solving the translational challenges of PC requires a forward-looking, multidisciplinary plan [20]. Because PC is so rare, advancing treatments depends entirely on global teamwork [88]. This includes sharing tumor registries and tissue samples across countries. Combining multi-omic data with advanced preclinical models will help researchers sort these complex tumors into treatable molecular groups. Moreover, unlocking the therapeutic efficacy of immunomodulatory agents remains a critical frontier. While immune checkpoint inhibitors (such as pembrolizumab) have revolutionized other cancers, a critical analysis of PC reveals that their broad application is limited by the predominantly “immune-ignorant” microenvironment of most parathyroid tumors [20]. Therefore, immunotherapy should not be applied empirically; rather, future perspectives on treatment must prioritize precision oncology, reserving checkpoint blockade strictly for the subset of PCs exhibiting high tumor mutational burden (TMB) or APOBEC-driven mutational footprints [20]. From a diagnostic perspective, the field must urgently transition from subjective morphological evaluations to objective, non-invasive modalities. The integration of artificial intelligence in digital pathology, paired with real-time liquid biopsy tracking of circulating markers like the BC200 transcript, represents the most promising avenue for improving early diagnosis and continuously monitoring therapeutic responses without repeated surgical interventions [54]. Finally, improving the prognosis for advanced PC requires a team effort. We must combine radical surgery with individualized, genotype-based drug treatments.

## Figures and Tables

**Table 1:** Key biomarkers for the diagnosis and prognosis of PC.

Biomarker (Gene)	Subcellular Localization	Expression Pattern in PC	Reported Sensitivity/Specificity (%) and Cohort Context	Clinical Utility and Significance	Level of Evidence	Key Reference(s)
Parafibromin (CDC73)	Nuclear	Complete or partial loss	68%/95% (Pooled meta-analysis of 202 PCs)	Surrogate Marker: Highly specific for CDC73 mutation; guides risk stratification and predicts recurrence.	Clinical (Meta-analysis/Retrospective Cohort)	Hu et al. [66]
Ki-67 (MKI67)	Nuclear	Elevated (>5% index)	45–60%/93–96% (Varies by cut-off criteria)	Core Prognosticator: High proliferative index strongly predicts aggressive clinical behavior and disease relapse.	Clinical (Retrospective Cohort)	Fernandez-Ranvier et al. [73], Uljanovs et al. [65]
Galectin-3 (LGALS3)	Cytoplasmic and Nuclear	Overexpression	45.4%/90.2% (Cohort of 14 PCs vs. 194 controls)	Diagnostic Adjunct: Enhances diagnostic specificity when incorporated into multimarker IHC panels.	Clinical (Retrospective Cohort)	Kumari et al. [69], Silva-Figueroa et al. [70]
PGP9.5 (UCHL1)	Cytoplasmic and Nuclear	Overexpression	45.4%/85.0% (Cohort of 14 PCs vs. 194 controls)	Diagnostic Adjunct: Overexpression strongly supports malignant diagnosis in morphologically challenging cases.	Clinical (Retrospective Cohort)	Kumari et al. [69]
Rb (RB1)	Nuclear	Loss of expression	33.3%/97.9% (Low sensitivity, high specificity)	Ancillary Indicator: Progressive loss of expression parallels the transition from adenoma to carcinoma.	Clinical (Retrospective Cohort)	Fernandez-Ranvier et al. [73]
E-cadherin (CDH1)	Membranous	Reduced or complete loss	47.7%/81.4% (Cohort of 44 PCs vs. 61 controls)	Invasion Marker: Reflects epithelial–mesenchymal transition (EMT), correlating with invasive growth potential.	Clinical (Retrospective Cohort)	Hu et al. [62]
EZH2 (EZH2)	Nuclear	Overexpression	31.8%/100% (Absent in 61 benign controls)	Epigenetic Discriminator: High-specificity indicator distinguishing PC from benign atypical neoplasms.	Clinical (Retrospective Cohort)	Hu et al. [24]
5-hmC	Nuclear	Complete loss	100%/100% (Highly specific cohort of 17 PCs)	Epigenetic Hallmark: Global loss rigorously correlates with malignant transformation and TET2 silencing.	Clinical (Retrospective Cohort)	Barazeghi et al. [71]
BC200 (BCYRN1)	Circulating (Serum)	Elevated	93%/75% (Evaluated via liquid biopsy)	Liquid Biopsy Tool: Non-invasive marker; tracks active tumor burden and monitors postsurgical response.	Clinical (Prospective/Retrospective Cohort)	Morotti et al. [54]

Abbreviations: PC, parathyroid carcinoma; IHC, immunohistochemistry; 5-hmC, 5-hydroxymethylcytosine; EMT, epithelial–mesenchymal transition. Note: Diagnostic performance data represent findings from specific study cohorts. Extreme metrics, such as 100% sensitivity or specificity, necessitate broad multi-center validation and must always be interpreted in strict conjunction with the histomorphological context.

## Data Availability

No new data were created or analyzed in this study. Data sharing is not applicable to this article.
